# Supplementary material for: Direct imaging of electron density with a scanning transmission electron microscope
Source: Nat Commun. 2023 Nov 20;14:7550. doi: 10.1038/s41467-023-42256-9 (PMC10662251; doi:10.1038/s41467-023-42256-9)
Supplement: Supplementary file 1 — Supplementary Information [file 41467_2023_42256_MOESM1_ESM.pdf]

Direct Imaging of Electron Density with a Scanning Transmission Electron Microscope

Ondrej Dyck[1§], Jawaher Almutlaq[2§], David Lingerfelt[1§], Jacob L. Swett[3], Mark Oxley,[1] Bevin Huang[2], Andrew R. Lupini[1], Dirk Englund[2], Stephen Jesse[1]*

[1] *Center for Nanophase Materials Sciences, Oak Ridge National Laboratory, Oak Ridge, TN, USA*

[2] *Massachusetts Institute of Technology, Cambridge, MA, USA*

[3] *Biodesign Institute, Arizona State University, Tempe, AZ, USA 87287*

[*]Corresponding Author e-mail: dyckoe@ornl.gov

[§]These authors contributed equally to this work.

Supplementary Information

Note 1. Misalignment

The WSe_2 and encapsulation layers are crystallographically misaligned/rotated as well as possessing different lattice parameters. This means that different positions for the W and Se-Se lattice sites in the WSe_2 will have different arrangements of atoms in the encapsulation layers. Supplementary Figure 1 illustrates a summary of these observations.

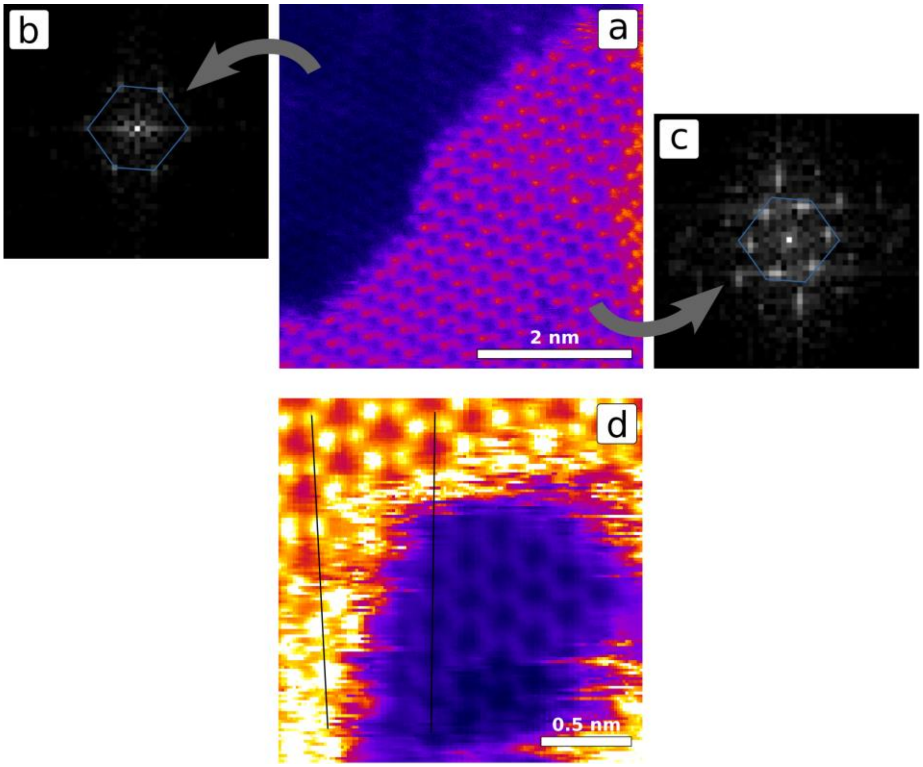

Supplementary Figure 1 Summary of crystallographic misalignment of the WSe_2 and encapsulation layers. (a) shows a HAADF image of the WSe_2 edge where the encapsulation layers extend beyond it. A fast Fourier transform (FFT) of this portion of the image is shown in (b) and the bright spots in the FFT have been traced with a hexagon. The FFT shown in (c) is taken from the WSe_2 portion of the image and the hexagon from (b) is overlaid. We observe a rotation and clear change in the dominant lattice parameter. (d) shows a magnified view of a portion of the heterostructure where the e-beam was in the process of milling through it. Vertical lines are aligned with features from the WSe_2 (left line) and h-BN (right line) illustrating the misalignment. The large difference in lattice parameter is also evident.

Note 2. Electron Energy Loss Spectroscopy

Because the SEEBIC signal is not generated from primary electrons, this signal can be simultaneously acquired with both the HAADF and EELS signals resulting in three distinct information channels. An example is shown in Supplementary Figure 2. (a) and (b) show simultaneously acquired HAADF and SEEBIC across a vacuum gap in the sample. Here, the WSe_2 layer is not present as determined using the HAADF image contrast. Using the SEEBIC image contrast we can see that the material at to top of the vacuum gap (top of the images) is not electrically connected to the TIA since it appears dark. But it is not clear whether the layers observed are h-BN or graphene. To make this determination we can use core-loss EELS. A second dataset was acquired collecting HAADF, SEEBIC, and EELS simultaneously, shown in Supplementary Figure 2(c)-(g). This dataset furnishes a one-to-one correspondence between each signal

channel and allows for a chemical analysis to be mapped across the sample. Here, we show a mapping of the total integrated core loss intensities from boron, (e), carbon, (f), and nitrogen, (g) core loss edges. Integrations were taken from the range marked 'Display Slice' in the spectra shown.

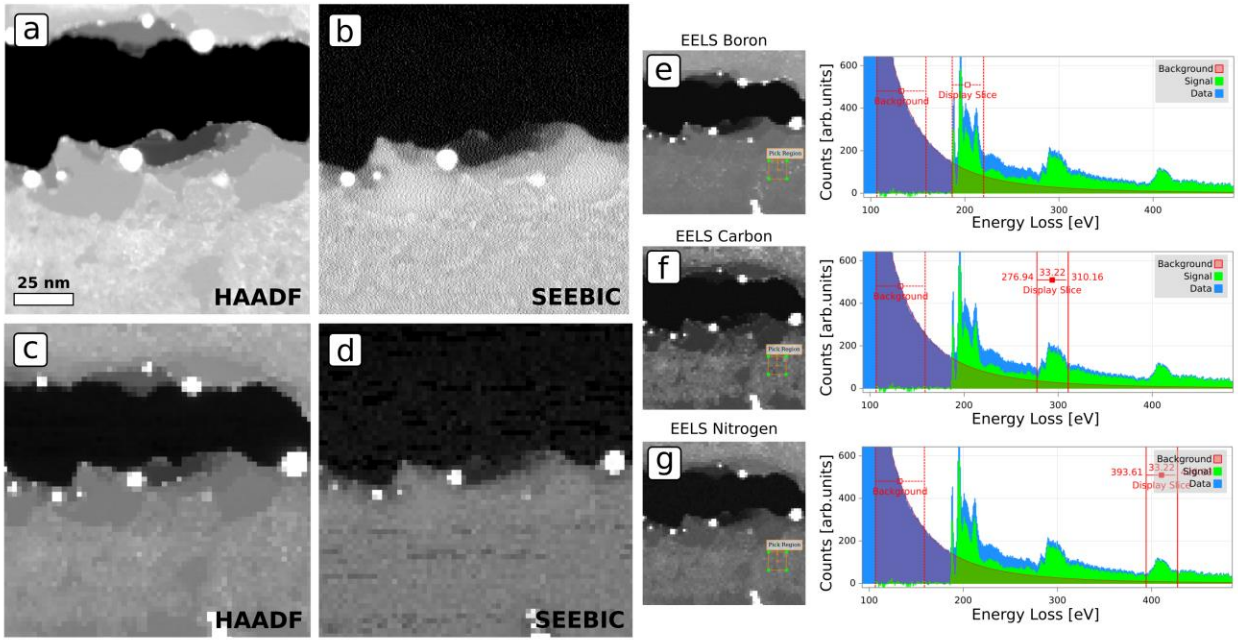

Supplementary Figure 2 Summary of simultaneously acquired HAADF, SEEBIC, and EELS data. (a) HAADF image across a vacuum gap in the sample. (b) Simultaneously acquired SEEBIC image. (c)-(g) Simultaneously acquired HAADF, (c), SEEBIC, (d), and EELS spectrum image (e)-(g). (e)-(g) show intensity maps generated by integrating the full data signal across the boron, carbon, and nitrogen core loss edges, respectively. Spectra on the right are averaged from the boxes indicated in the images. Raw data and background subtracted data are overlaid in blue and green, respectively.

Note 3. TD-DFT Simulations of Secondary Electron Generation

This section provides details of our time-dependent density functional theory simulations of secondary electron generation via inelastic scattering. In order to assess the total secondary electron (SE) yield resulting from electron beam irradiation at a particular position within the material, we apply a beam-like perturbation to our model system (isolated clusters of WSe_2), impose absorbing boundary conditions during the ensuing electronic dynamics, and monitor the charge density removed from the system in the long simulation time limit as a measure of the secondary electron yield. References 1 and 2 describe closely related approaches for simulating secondary electron emission that have been applied previously, where the

velocities of the primary electrons/ions were significantly lower than those of the 80+ kV electron beams (relativistic velocities over half the speed of light) used in the current work. Explicitly simulating the time-dependent response to such high-velocity incident electrons would require an impractically small TD-DFT time step to ensure a converged/consistent result. Accordingly, we instead rely on an approximate approach that was previously developed by the authors, which considers the electronic (linear) response of materials to an impulse perturbation determined by the *average* potential experienced by the system during the passage of an (undeflected) electron. Indeed, the principal difference between our current approach and that adopted by Kononov et. al.[2] (notwithstanding any technical differences in the implementation of TD-DFT and absorbing boundary conditions) lies in how the electric potential associated with the external charged particles is incorporated into the time-dependent electronic Hamiltonian.

Our approach employs time-dependent density functional theory calculations within the "real-time" propagation approach, which solves the time-dependent Kohn-Sham equations for the evolution of a system's electronic state as an initial value problem. All TD-DFT calculations in this study were initialized in the electronic ground state prior to the application of the beam-like perturbation, and used the self-consistent second order Magnus integrator implemented in NWChem with a time step of 0.2 atomic units (~4.8 as) along with a power series expansion approach for matrix exponentiation operations. The average scalar potential of a line of equally (0.1 Bohr) spaced electrons (represented as point charges) oriented normal to the basal plane of the material was used to perturb the system at different positions in the imaging plane. Only the potential contributed by beam electrons on approach to the center of the material was included, so as not to preclude the excitation of out-of-plane polarized transitions in this symmetric system. A complex absorbing potential was imposed during the ensuing dynamics, imparting a brief lifetime (exponential time-constant of 1.0 atomic units; default value in NWChem) to electrons residing in unbound orbitals that results in the eventual removal of their contribution to the charge density. The system's first vertical ionization energy was approximated by way of Janak's theorem. Further details of the absorbing potential implemented in the NWChem TD-DFT module can be found in Ref. 3.

Here, we briefly demonstrate our approach on a series of simple atomic test systems (helium, neon, argon, and krypton; Figure S3) using a large basis (aug-cc-pvtz) and 100% exact exchange. Already for the heavier atomic species, we can appreciate the emergence of fine structure in the position-dependent SE yield, that reports on the local density of states. This nontrivial position dependence is precisely the aspect of the SE yield that we are aiming to resolve under the current approach. Integrating these ionization rates (scaled by the appropriate volume element) gives a total SE yield for the atomic species evaluated within our model. The relative trend in these total SE yields across the different elements agrees qualitatively with that from

the (experimentally corroborated) theoretical total ionization cross sections obtained for high-energy incident electrons within the binary encounter dipole approximation (see, for instance, Ref. 4)

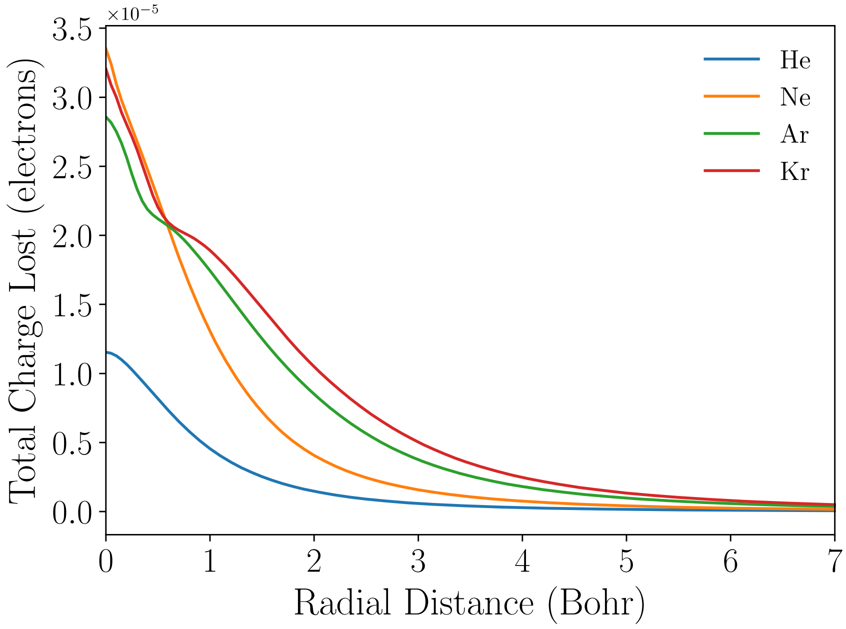

Supplementary Figure 3. Total charge lost to the absorbing boundary conditions after acting our beam-like perturbation at a given distance from the center of mass of neutral helium, neon, argon, and krypton atoms. Simpson's rule numerical integration of these quantities (scaled by the volume element) gives a ratio for the total SE yields within our model of 1:2.76:5.05:6.43 for He:Ne:Ar:Kr.

While our approach has been specifically designed to give a direct approximation to the position-dependence of secondary electron yields due to high-energy electron beam irradiation, the approximations involved significantly limit its range of applications. For instance, we have no mechanism to capture the beam energy-dependence of the ionization cross section under this approach, which would require using the relativistically-correct electromagnetic (Lienard-Wiechert) matter-beam interaction potential and also an explicitly time-dependent description of the interaction during the scattering dynamics. There is also no description of (multiple) scattering effects on the probe function, which we represent in our simulations as perfectly columnated with no spread in the imaging plane. Note, also, that while the escape depth for a SE is neither defined nor imposed explicitly in our approach, electrons ejected from atoms residing deeper in the interior of the material have the opportunity in our simulation to interact with the material during their migration towards the vacuum and be recaptured.

Finally, to illustrate the effects one can expect from including the laminating hBN layers

in our simulations, we have simulated the SE yield for the WSe_2 cluster model system presented in the main article, but in close proximity to an arbitrarily-aligned hydrogen-terminated hBN overlayer (with distance between W and B/N atomic planes taken to be the mean interlayer distance of the two materials, or 3.88 Angstrom). The charge lost by the WSe_2 layer is resolved along one of the W-Se bonding directions using an analogous approach to that employed for the simulations in the main article. As in the results featured in the main article, the beam-like perturbation extends only outward from the W atomic plane, and for the current simulations in the direction opposite the hBN layer. The goal of this computational experiment is to demonstrate modulation of the SE yield from the WSe_2 layer due to the presence of the nearby hBN layer. We approximate the SE from the WSe_2 component of the bilayer system by first evaluating the total charge lost through TD-DDT simulation of the full system, and then subtracting away the charge lost from analogous simulations employing the same perturbing potentials with the WSe_2 component deleted. In this way, we aim to resolve the influence of the hBN overlayer on the WSe_2 ionization, and avoid the more trivial result that the total secondary electron yield is increased in the heterobilayer system by the direct ionization of the hBN. The results (Supplementary Figure 4) indicate that SE yield is modulated to a different extent by the presence of the hBN layer for the different positions of the beam-like perturbation, and thus suggest that the variations in SEEBIC intensity for identical lattice sites of the embedded WSe_2 layer resulting from electronic interactions with encapsulating materials (with different periodicities than the embedded layer) report in some capacity on the atomistic details of the materials' interface.

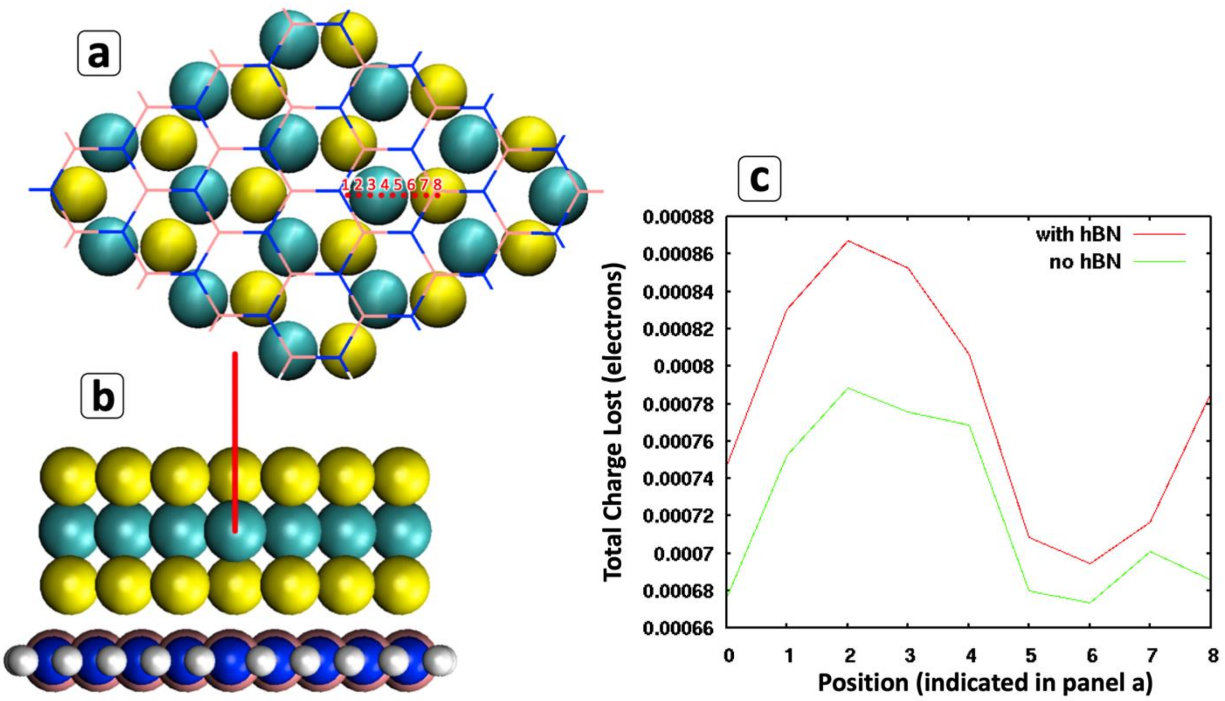

Supplementary Figure 4. Simulated secondary electron yields for a series of different real-space beam-like perturbations applied to a simple WSe_2 and hBN heterobilayer model system. For each of the positions indicated by numbered red points in (a), separate TD-DFT electronic dynamics simulations were carried out after subjecting the system to an electric field impulse defined by the average electric potential associated with a line of 100 evenly spaced electronic point charges extending 10 Bohr radii from the indicated positions (in the W atomic plane), as illustrated in (b). The total charge lost to the absorbing boundary conditions upon perturbing at each position indicated in (a), less the charge lost from an identically prepared TD-DFT simulation with the WSe_2 cluster removed, is plotted in panel (c) labeled 'with hBN', along with the analogous data from the isolated WSe_2 cluster labeled 'no hBN'.

Supplementary References

(1) Ueda, Y.; Suzuki, Y.; Watanabe, K. Quantum Dynamics of Secondary Electron Emission from Nanographene. *Phys. Rev. B* **2016**, *94* (3), 035403. https://doi.org/10.1103/PhysRevB.94.035403.

(2) Kononov, A.; Lee, C.-W.; dos Santos, T. P.; Robinson, B.; Yao, Y.; Yao, Y.; Andrade, X.; Baczewski, A. D.; Constantinescu, E.; Correa, A. A.; Kanai, Y.; Modine, N.; Schleife, A. Electron Dynamics in Extended Systems within Real-Time Time-Dependent Density-Functional Theory. *MRS Communications* **2022**, *12* (6), 1002–1014. https://doi.org/10.1557/s43579-022-00273-7.

(3) Lopata, K.; Govind, N. Near and Above Ionization Electronic Excitations with Non-Hermitian Real-Time Time-Dependent Density Functional Theory. *J. Chem. Theory Comput.* **2013**, *9* (11), 4939–4946. https://doi.org/10.1021/ct400569s.

(4) Bartlett, P. L.; Stelbovics, A. T. Calculation of Electron-Impact Total-Ionization Cross Sections. *Phys. Rev. A* **2002**, *66* (1), 012707. https://doi.org/10.1103/PhysRevA.66.012707.
